# Forequarter amputation post transarterial chemoembolization and radiation in synovial sarcoma: A case report

**DOI:** 10.1016/j.ijscr.2021.105824

**Published:** 2021-03-23

**Authors:** Erwin Danil Yulian, Jacub Pandelaki, Evelina Kodrat, I. Gusti Ngurah Gunawan Wibisana

**Affiliations:** a Surgical Oncology Division, Department of Surgery, Dr. Cipto Mangunkusumo General Hospital, Faculty of Medicine Universitas Indonesia, Indonesia; b Interventional Radiology Division, Department of Radiology, Dr. Cipto Mangunkusumo General Hospital, Faculty of Medicine Universitas Indonesia, Indonesia; c Department of Anatomical Pathology, Dr. Cipto Mangunkusumo General Hospital, Faculty of Medicine Universitas Indonesia, Indonesia

**Keywords:** TACE, transarterial chemoembolization, SS, synovial sarcoma, EMA, epithelial membrane antigen, SMA, smooth muscle actin, Forequarter amputation, Transarterial chemoembolization, Radiation, Synovial sarcoma, Case report

## Abstract

**Introduction and importance:**

Forequarter amputation or interscapulathoracalis amputation is a major amputation procedure that involves the entire upper extremity, scapula, and a whole or part of the clavicula. Forequarter amputation is commonly used to control bleeding in malignant tumor cases in which no treatment is available for the extremities.

**Case presentation:**

We report a case of forequarter amputation in a 25-year-old patient with synovial sarcoma. Transarterial chemoembolization (TACE) and radiation synovial sarcoma were performed in the patient to reduce bleeding. This technique may also be used for treating synovial sarcoma with massive bleeding.

**Clinical discussion:**

Despite forequarter amputation indications in malignant tumor cases and recurrent cancer cases, the effectiveness of this technique remains unclear. The patient was readmitted with a recurrent mass three months after surgery.

**Conclusion:**

In this study, TACE and radiotherapy are effective in controlling bleeding preoperatively and intraoperatively in patients with synovial sarcoma.

## Highlights

• Forequarter Amputation is a safe and feasible option for patients with soft tissue malignancy like synovial sarcoma.
• Forequarter Amputation has a beneficial impact on the patient’s psychological and functional integrity.
• Transarterial Chemoembolization and radiotherapy can be the treatment option for the patient with malignant bleeding.
• TACE and radiotherapy are effective in controlling the pre-operative and intraoperative bleeding of forequarter amputation.
• The TACE procedure aimed to control bleeding, strengthen local surgical control and decrease the morbidity of wide excisions.

## 1 Introduction

Synovial sarcoma (SS) is a malignant soft tissue tumor with high recurrence and distant metastases. SS accounts for 5–10% of all forms of soft tissue tumors, which usually affects adolescents and young adults [1,2]. Sarcoma of this type occurs mostly in the upper part of the knee and in the parapharyngeal region of the head and neck [[1], [2], [3]]. It has often been identified in periarticular regions, close to tendon sheaths, bursae, and joint capsules. In general, SS is circumscribed, but it may also be entangled with muscles, tendons, and neurovascular structures around it in a few instances. The lungs, lymph nodes, liver, and bones are the most common sites of metastasis. SS has a poor prognosis, which lies between 36 and 76 percent and 20 and 63 percent for 5- and 10-year survival rates, respectively. Wide surgical resection, with or without radiotherapy, is the preferred treatment for SS. Limb-sparing surgery is commonly performed in soft tissue sarcoma, but extremity amputation may be used as a curative and palliative treatment for malignant tumors [[4], [5], [6], [7]]. Forequarter amputation is a supportive treatment in the shoulder girdle of patients with soft tissue sarcoma [6]. Only a few cases of malignant tumors with nerve and vein infiltration in the shoulder girdle have been reported that require this type of amputation to curatively and palliatively achieve oncological margins [6,8].

Forequarter amputation was first performed in 1808 for treating a bullet wound and first used to treat upper extremity malignancy in 1836 [9]. It is a radical ablative surgical operation involving the full upper extremity of the shoulder girdle [10]. The indications of this treatment are persistent severe pain, neurovascular involvement, major primary tumor development, failure to maintain limb function with full tumor margin resection, inadequate chemotherapy, and radiotherapy, high-grade sarcoma pathological fracture, severe lymphedema, bleeding, and palliative therapy [[9], [10], [11]].

Preoperative embolization for controlling blood loss in intraoperative and postoperative settings has been reported for use in conjunction with sarcoma resection. However, its use in preoperative amputation has not been described. Transarterial chemoembolization (TACE) is performed before surgery to control the hemorrhage [10,11]. Embolization in the primary soft tissue tumor or metastatic embolization in the extremities is indicated to reduce hemorrhage risk during and after surgery. The primary purpose of embolization is to achieve thrombus formation and arterial occlusion by administering embolizing agents using a selective or super-selective catheter inserted into the feeding arteries of the tumor. This case report presents a patient with recurrent synovial sarcoma of the upper extremity who was treated with radiotherapy to control hemorrhage, followed by preoperative embolization before forequarter amputation. The work has been reported in line with the SCARE 2020 criteria [12].

## 2 Case presentation

**Fig. 1:**
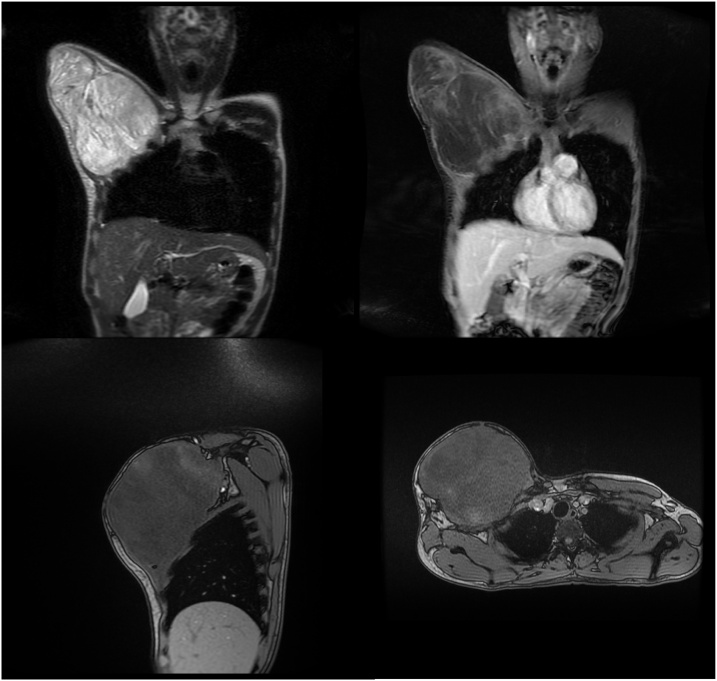
MRI results showing recurrent tumor.

A 24-year-old man was admitted to the ER with a large, painful (VAS 8), and recurrent bleeding mass in the right upper chest quadrant, increasing in size for two months before admission. Before the surgery, the mass dimension was 14 cm × 9 cm × 6 cm, with the pathological result of a grade II myxofibrosarcoma. The patient did not suffer from cough, dyspnea, or fever. There was no family history of malignancy in the family. MRI (Fig. 1) and CT Scan (Fig. 4) showed that the patient had a recurrent tumor.

The histology slides were examined, and the morphological features showed spindle cell sarcoma reminiscent of low-grade fibromyxoid sarcoma with extensive necrosis. Immunohistochemical staining was not entirely specific due to extensive necrosis, but as a low-grade fibromyxoid sarcoma, the morphology could still be supported [13]. We performed wide surgical excision with removal of the pectoralis major muscle and obtained a primary tumor measuring 22 × 13 × 18 cm with a non-tumor-free margin. A high-grade sarcoma of the spindle cell was the postoperative pathological outcome. Immunohistochemical staining revealed epithelial membrane antigen (EMA) focal positivity, and CD99, BCL2, and CD56 positive, smooth muscle actin (SMA) negative, and desmin and S100 protein-positive results. This staining pattern was mostly associated with monophasic synovial sarcoma along with the morphology [13]. We then performed a PET scan with no tumor cells. Thereafter, the patient refused to receive radiation and chemotherapy for 2 months and chose to be treated by a medicine man for two months rather than receiving chemotherapy. Meanwhile, the mass reappeared in the right upper quadrant of the chest and in the right upper extremity, which had been growing in size as large as an adult head. The patients suffered from a swollen right hand and active hemorrhage in the masses (Fig. 1). Multiple blood transfusions were needed, and several treatments, including corticosteroid injections, betadine dressing, flamazine, and cauterization, were considered to stop the bleeding. Hemostasis was achieved only by compression bandage, and bandage removal induced rebleeding. We performed external radiation for hemostatic purposes six times, which resulted in reduced hemorrhage. However, after the external radiation procedure, bleeding of the mass was still ongoing. Because of the large tumor size and extensive vascular and soft tissue involved, we planned a salvage surgery rescue procedure. PET did not reveal any other metastatic lesions.

After extensive consultations, a multidisciplinary meeting consisting of oncology surgeons, cardiothoracic and vascular surgeons, pathologists, and radiation oncologists agreed that amputation was the best alternative to treat pain, bleeding, lymphedema, and compromised patient function. A team of oncology surgeons and cardiothoracic and vascular surgeons performed the procedure. Based on a discussion with the patient and his family about the procedure’s radical nature, benefit, and potential complications, they agreed to undergo the operation. He had restricted arm mobility due to mass impact, chronic intractable pain, and lymphedema.

**Fig. 2:**
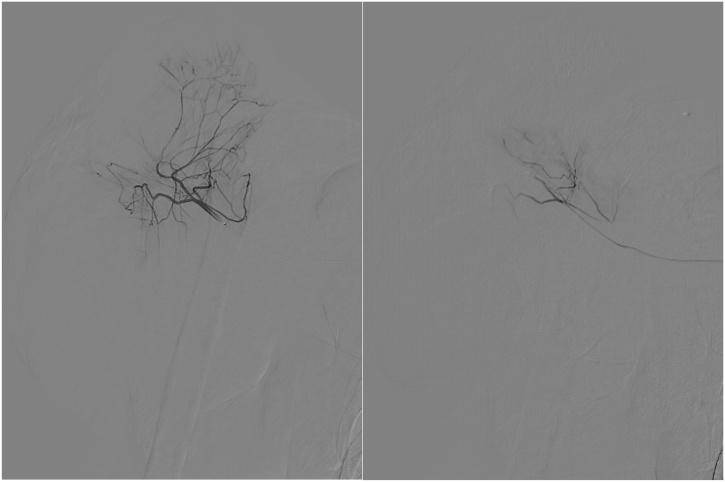
Digital subtraction angiography of axillary artery; pre-embolization (left) and post-embolization (right).

Preoperative embolization was advised by interventional radiologists during preoperative consultation. Due to the large tumor size at the proximal area and a previously irradiated bed mass position, we preserved the vascular function (subclavian and axillary vessels) and reduced wound bleeding during surgery. TACE was performed in the tumor artery, and the patient underwent right subclavian and axillary artery angiography with embolization of the right subclavian artery (Fig. 2). The TACE procedure aimed to control bleeding, strengthen local surgical control, and decrease the morbidity of wide excisions.

Vascular access from the right femoral artery was achieved with introducer sheath 6 Fr, and a diagnostic catheter 5 Fr was inserted until the tip reached the right subclavian artery. This was continued with the mapping arteriography. Hypervascularization of the feeding artery originating from the superolateral right axillary artery neovascularization branch and the proximal inferomedial right brachialis artery neovascularization branch was demonstrated by this procedure. Then, microcatheter 2,4 Fr was inserted until the tip was selectively located in the neovascularization of these branches, and a chemoembolization agent was inserted. GS particles and microcoils were used in this procedure. The chemoembolization agent used in this procedure was doxorubicin (50 mg) and Lipiodol (10 mL), including the embolization agent, PVA (500–700 microns), and gel foam combination. The operation was performed from the neovascularization branch of the superolateral right axilla artery in the feeding artery and the proximal inferomedial right brachialis artery in the neovascularization branch.

**Fig. 3:**
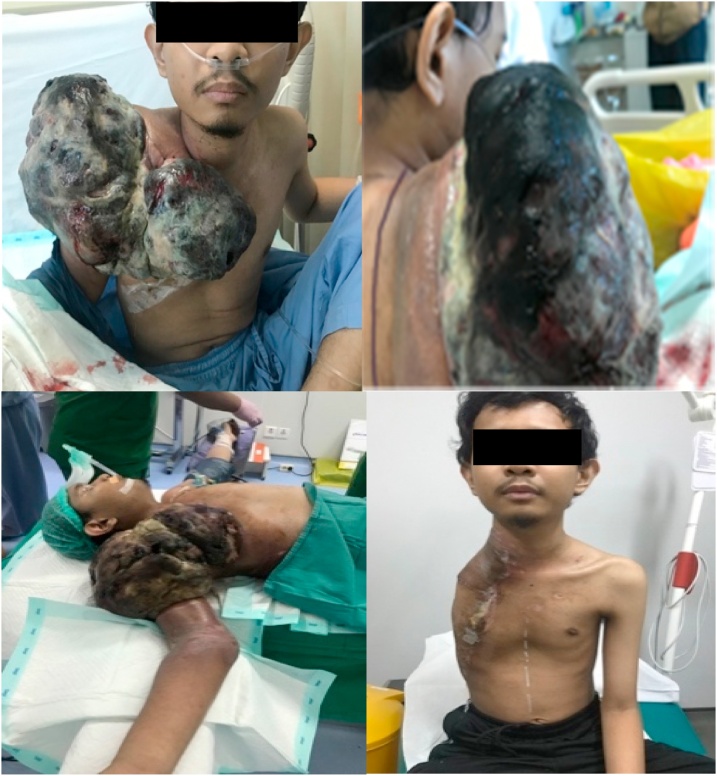
Preoperative, intraoperative, and postoperative of forequarter amputation.

Six days after the TACE procedure, we performed extensive surgery on the entire right upper extremity, scapula, and whole clavicula with 235 mL bleeding during the surgery, and the size of the mass was 30 × 24 × 10.5 cm from the right upper quadrant of the chest and shoulder girdle (Fig. 3).

**Fig. 4:**
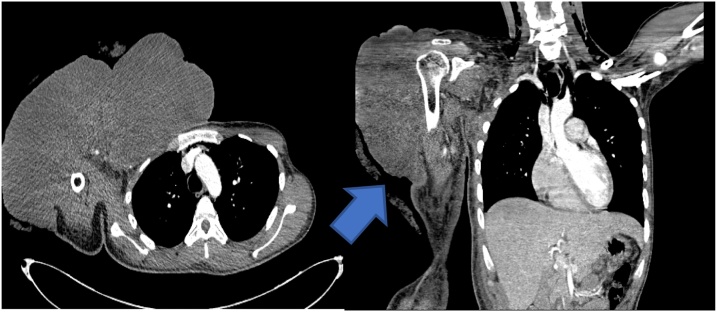
Thorax CT scan showing the tumor mass.

## 3 Surgical procedure

Under general anesthesia, surgery was performed with the patient in the full lateral position. Several surgical techniques have been described for performing a forequarter amputation. We used a semi-lateral approach, and the majority of the surgery was performed anteriorly. The patient was in a left lateral decubitus position to reveal the thorax, with the right upper extremity positioned to rotate freely. We made an elliptical incision on the superior apex above the clavicle at one end and proceeded inferiolaterally. Skin flaps were elevated to the anterior midline, superior to the clavicle, inferior to the costal arch, and medial to the midline. The latissimus dorsi and the superomedial border of the mass were the lateral borders of the skin flaps. At the origin of the sternum, we dissected the pectoralis major muscle from the medial as the insertion, which was also invaded by the mass. On the anterior side, the pectoralis muscle was infiltrated by the tumor mass, separating the pectoralis major and minor.

The brachial plexus and axillary vessels were exposed anteriorly. The surgical technique involved an incision on the preoperative markings at a distance of 2 cm from the edge of the induration. We then performed anterior vascular exploration by separating the major pectoralis muscle from the clavicle. These were deepened into the musculofascial layers. The platysma and supraclavicular nerves were excised. To allow control of the subclavian vessels, clavicular osteotomy was performed by exposing the clavicle and cutting laterally to the sternocleidomastoid with a Gigli saw. The tendons of the pectoralis major, coracobrachialis, and the short head of the biceps were cut. Dissection was continued to the thoracic wall in order to excise the pectoral muscles, soft tissues, and the ribs’ periosteum. First, ligation of the subclavian artery was performed, and the artery was cut. Thereafter, the subclavian vein was ligated and cut. This was performed to avoid any bleeding from the shoulder’s collateral blood supply. The brachial plexus was proximally ligated and divided.

A posterior incision was made, all periscapular muscles were released, and fasciocutaneous skin flaps were developed. The major and minor rhomboid, trapezius, omohyodeus venter inferior, and levator scapulae muscles were then split by electrocautery as they were inserted into the scapula. Moving caudally, we transected the latissimus dorsi and resected it from the chest wall. The exposure was continued to the rhomboid muscle insertions posteriorly and laterally. An axillary incision was made to connect the anterior and posterior incisions. This freed the shoulder girdle and the entire extremity from the trunk. Finally, after the subclavian artery and brachial plexus ligation and transection, the entire forequarter was removed, along with the tumor material, including the scapula, inevitably producing a large tissue defect. Posterior skin flaps were used to close the defect. The dog-ear skin excess was excised. Finally, drains were used to prevent the development of hematoma and wound complications.

No significant complications of forequarter amputation were observed, such as the development of a pneumothorax with consecutive respiratory insufficiency. Our patient complained of the minor complication of delayed wound healing, which required local surgical debridement. He also suffered from postoperative phantom pain or local pain with moderate VAS scores of 5–6 that could be overcome with oral analgesics. When the wound healed after two weeks, he was released from the hospital.

The pathology findings were consistent with the previous diagnosis of monophasic synovial sarcoma [15]. Three weeks after the forequarter amputation, the patient underwent 30 rounds of radiation therapy. Three months after the surgery, PET showed no tumor mass. Two weeks after the last PET scan, two masses reappeared on the right hemithorax near the amputation wound. Surgical excision of 1.2 × 0.8 × 0.8 cm mass with pathological outcome of monophasic grade IIII synovial sarcoma was performed. One week after the last surgical excision, the patient had to undergo chemotherapy with regimen of doxorubicin, mesna, and holoxan.

The patient seldom experienced phantom limb pain. This is a common sequela of forequarter amputations. The patient received 4 out of 6 cycles of chemotherapy, which was safe. Due to the Covid-19 pandemic, the patient was afraid to go to the hospital. Eleven months after the procedure, the patient died of respiratory failure in his home.

## 4 Discussion

Forequarter amputation is an ablative and radical surgery to remove the entire upper extremity, including the shoulder girdle [10]. Amputation has been considered as the standard of care for patients with soft tissue or bone sarcoma of the limbs. The general aim of this procedure is to radically remove soft tissue and bones, including the scapula. Even though the result is disfiguring, as observed with our patient, forequarter amputation is able to effectively palliate the upper extremity tumor [10]. The indications of forequarter amputation are for limb-sparing resection, unresectable sarcoma, recurrent soft tissue sarcomas following an ineffective limb-sparing operation, several shoulder girdle radiation-induced sarcomas, pathological high-grade sarcoma fractures (particularly for poor response to induction chemotherapy), and palliative amputation (due to fungating tumor, infection, or bleeding) [14,15]. Amputation in the forequarters has a beneficial impact on the patient’s psychological and functional integrity. Elsner et al. [16] revealed that forequarter amputation remained a reasonably safe and effective technique for upper extremity malignancy curative or palliative care, especially in the absence of other less radical alternatives [10,16]. Daigeler et al. [17] found that patients treated with proximal major amputation had an increased quality of life and post-amputation pain relief. The study also reported a correlation between full resection and prolonged survival, considered to localize the disease with curative purpose [17]. In patients who had recurrent tumors after amputation, remote and occult metastases were usually present. However, there was no significant difference in patient survival time between the disseminated disease and the localized disease at the time of the amputation [17]. Wittig et al. [18] found that postoperative survival ranged from 3 to 12 months in patients with palliative forearm amputation. We report a challenging case of a large and localized shoulder synovial sarcoma. With the recent availability of chemotherapy and radiotherapy, the primary aim of our treatment was limb-sparing resection as one of the indications of forequarter amputation [14]. The tumor must be completely excised to retain limb function while the brachial plexus is maintained for the patient [14]. Therefore, we performed forequarter amputation surgery on this patient. We believe that this procedure was best tolerated by our patient, who suffered intractable pain and a dysfunctional limb.

Forequarter amputation is a complicated procedure with physical and mental effects due to total functional loss of the limbs and significant optical disfigurement. Locally advanced tumors spreading to the axilla represent indications for forequarter amputation and become irresectable with a limb-sparing procedure [19]. By etiology, after a failed limb-sparing operation, these tumors are most commonly high-grade shoulder soft tissue sarcomas, axillary soft tissue sarcomas affecting recurrent bone, brachial plexus, and soft-tissue sarcomas [20]. Severe untreated pain, total functional failure of the affected leg, and not infrequently pronounced lymphedema, are the major candidates for sequential amputation. Palliation, even though only briefly accomplished, becomes an even more critical feature in this case [20].

In the present case, the patient experienced active bleeding from the tumor mass. Bleeding complications typically arise in patients with advanced cancer, with 10% experiencing at least one episode of bleeding [21,22]. Bleeding from cancer, such as local tumor invasion, irregular vasculature of the tumor, tumor regression, or systemic coagulopathy, is induced by paraneoplastic syndrome [22,23]. Bleeding in cancer patients may be worsened by the use of immunotherapy, nonsteroidal anti-inflammatory drugs (NSAIDs), and anticoagulants. To avoid bleeding at the tumor site, anti-inflammatory medication for pain in advanced cancer needs to be reconsidered [22]. Local alternative therapies, including dressing, pressure application, packing, radiation therapy, percutaneous embolization, and surgery recommended [22].

In neurosurgery, urology, general surgery, head and neck surgery, orthopedics, and oral surgery, preoperative embolization has been identified as essential to reducing intraoperative bleeding. Despite potential complications, including arterial infarction, ischemic event, nerve injury, and local hemorrhage, most have been successfully performed [9]. In patients with renal cell carcinomas, a tumor embolization technique has been developed [19]. Minimal invasion of transcatheter arterial embolization was helpful in achieving hemostasis when surgical ligation was difficult [24].

Reducing the risk of bleeding postoperatively for hypervascularized tumors, simplifying tumor manipulation, increasing the chemotherapy response and radiotherapy, and palliative care of pain are the main indications for embolization. When surgery is inappropriate or associated with a higher risk, embolization can also be a therapeutic option for surgery [25]. Moreover, the distinctions between the tumor and the surrounding tissue are becoming clearer, making it easier for operational manipulation and excision. Tumor-feeding arteries must be super-selectively catheterized, and the most effective embolizing agents should be used to preserve the hemodynamics of the normal surrounding tissues [25].

When performed by an experienced team with proper embolizing agents, preoperative selective/superselective transarterial embolization of hypervascular malignant soft tissue tumors of the extremities is an efficient and safe procedure. To avoid complications, achieve successful devascularization, and complete resection of a tumor, super-selection and flow control are essential [25]. A related study by Iwamoto et al. [24] revealed an ideal indication for arterial embolization prior to musculoskeletal tumor excision to prevent significant intraoperative bleeding, cases of low risk of serious complication following embolization, and cases of trunk, pelvic, and proximal limb hypervascular tumors, except for the peripheral and spinal areas. In a study conducted by Iwamoto et al., the use of selective transcatheter arterial embolization for musculoskeletal bone tumors significantly reduced intraoperative bleeding after arterial embolization. A retrospective analysis by Jiang et al. [26,27] found that cancer pain (as measured by VAS) was decreased after the TACE procedure in patients with advanced soft tissue sarcoma, but cancer pain was more common in the chemoembolization group than in the chemo infusion group. Jiang et al. [26] also reported that patients with soft tissue sarcoma had a 32.5% overall 3-year survival, but this did not mean that the overall survival of the chemoembolization group was higher than that of the chemotherapy infusion group. However, in patients who underwent active chemoembolization, the mean relapse time was longer than in patients who only underwent chemo-infusion. Jiang et al. also found that the efficacy was affected by the PVA diameter. In patients with a PVA community diameter of 100 μm, the mean relapse period was longer than 300 μm [26].

Synovial sarcoma (SS) is an active tumor of soft tissue with a propensity for local recurrence and a high risk of distant metastasis. Unlike many other sarcomas, in only 10–20 percent of cases, it may invade the adjacent bone. With 5- and 10-year survival rates of 36–76 percent and 20–63 percent, respectively, the prognosis for SS is usually poor. Under 20 years of age, lower tumor stage, tumor size < 5 cm, sufficient excision, and a more distal position of the extremities are factors associated with better prognosis. Poor prognosis predictors include less-differentiated tumor regions, necrosis, and high mitotic activity [28]. Bleeding is a common concern in patients with advanced cancer and can occur in 6–10% of advanced cancer cases; a few of these cases may be the direct cause of death for at least one episode. Patients and their families are distressed; bleeding is also likely to have a negative effect on the quality of life of the patient (QoL) [29]. In patients with advanced malignancy, multiple modalities are conventionally used to control hemorrhage, as reviewed by Pereira and Phan, including hemostatic agents, endoscopy, vessel ligation, cauterization, tissue resection, transcutaneous arterial embolization, balloons placements, and radiotherapy. Blood products, vasopressin, antifibrinolytic agents, and somatostatin analogs are also systemic therapies [23].

For decades, radiotherapy (RT) has been used as a non-invasive treatment for bleeding associated with cancer. After only a few fractions of RT, the hemostatic efficacy of radiotherapy is normally visible and is commonly clarified by increased platelet adhesion to the vascular endothelium. By inducing vessel fibrosis combined with tumor remission, the long-term effect could be clarified [29]. Although the precise mechanism to manage malignant bleeding remains unclear, radiotherapy is used to control bleeding in some types of cancer. RT destroys the malignant blood vessels. By causing malignant endothelial cell damage, radiation can induce the pathophysiological processes of malignant vessels. The signal transduction pathway, which leads to cell cycle arrest, can also be affected. Tumor bleeding can also be caused by vascular damage due to local tumor invasion or systemic coagulopathy due to paraneoplastic syndrome. RT may not, however, prevent bleeding of normal vessels that are invaded by cancers, such as carotid blowouts in progressive malignancy of the head and neck [23].

The pathophysiology of the cancer radiation effect causes significant damage to DNA by inducing malignant damage to endothelial cells, causing signal transduction pathway activation and leading to cell apoptosis [23]. Radiotherapy was shown to have an effect within 24−48 hours of the first dose. There were several palliative care regimens available for patients with cancer bleeding, including single 8–10 Gray (Gy) procedures, intermediate 4–8 Gy implementation 3–5 times, or longer 30–45 Gy implementation 10–15 times [22]. The improved convenience and reduced cost approach was reported by Van den Hout et al. in 91 percent of advanced and metastatic cancer patients with shorter radiation treatments [30]. A retrospective analysis of 62 patients with advanced cancer found malignant bleeding could be reduced by hemostatic radiotherapy [29]. Sapienza indicated that radiotherapy could treat bleeding, resulting in an 89 percent overall primary bleeding control rate. By position, 88 percent (14/16 for head and neck), 93 percent (13/14 for thoracic), 100 percent (9/9 extremity), and 100 percent (6/6), and gynecologic sites were the primary bleeding control rates. While the effect of hemostatic radiotherapy has not been sufficiently evident in synovial sarcoma, it has the potential to control bleeding in other malignancies [31].

Research by Sapienza et al. has shown that palliative radiotherapy is efficient in primary bleeding control [31]. Nevertheless, longer radiotherapy regimens, which were more than 5 fractions, resulted in an increased length of hospital stay and side effects. Hence, in this palliative setting, shorter hemostatic regimens are preferred to minimize the care burden for patients. Administering a higher dose should consider the balance between the possible benefits and potential toxicity. In the event of life-threatening bleeding, hypofractionation with a large single dose can induce rapid hemostasis, but even in this palliative end-of-life environment, the risks of extreme toxicities must be considered [29].

## 5 Conclusions

For patients with soft tissue malignancies such as synovial sarcoma, particularly tumor recurrence with bleeding manifestation, forequarter amputation is a safe and reliable treatment option. TACE and radiotherapy can be treatment options for patients with malignant bleeding and are effective in controlling preoperative and intraoperative bleeding during forequarter amputation.

## Declaration of Competing Interest

The authors report no declarations of interest.

## Sources of funding

None declared.

## Ethical approval

Not applicable.

## Consent

Written informed consent was obtained from the patient for publication of this case report and accompanying images. A copy of the written consent is available for review by the Editor-in-Chief of this journal on request.

## Authors contribution

**Erwin Danil Yulian**: Concept and design of case report, data collection, drafting, revision, approval of final manuscript.

**Jacub Pandelaki**: Data collection, revision, approval of final manuscript.

**Evelina Kodrat**: Data collection, revision, approval of final manuscript.

**I Gusti Ngurah Gunawan Wibisana**: Data collection, revision, approval of final manuscript.

## Registration of research studies

Not applicable.

## Guarantor

Erwin Danil Yulian.

## Provenance and peer review

Not commissioned, externally peer-reviewed.

## Presentation at a meeting

None declared.

## References

[1] Goldblum J.R., Folpe A.L., Weiss S.W. (2019). Soft Tissue Tumors.

[2] Krieg A.H., Hefti F., Speth B.M., Jundt G., Guillou L., Exner U.G. (2011). Synovial sarcomas usually metastasize after &5 years: a multicenter retrospective analysis with a minimum follow-up of 10 years for survivors. Ann. Oncol..

[3] Machen S.K., Easley K.A., Goldblum J.R. (1999). Synovial sarcoma of the extremities: a clinicopathologic study of 34 cases, including semi-quantitative analysis of spindled, epithelial, and poorly differentiated areas. Am. J. Surg. Pathol..

[4] Lehnhardt M., Hirche C., Daigeler A., Goertz O., Ring A., Hirsch T. (2012). Soft tissue sarcoma of the upper extremities: analysis of factors relevant for prognosis in 160 patients. Chirurg.

[5] Robert R.S., Ottaviani G., Huh W., Palla S., Jaffe N. (2010). Psychosocial and functional outcomes in long-term survivors of osteosarcoma: a comparison of limb-salvage surgery and amputation. Pediatr. Blood Cancer.

[6] Clark M.A., Thomas J.M. (2003). Major amputation for soft-tissue sarcoma. Br. J. Surg..

[7] Ottaviani G., Robert R.S., Huh W.W., Jaffe N. (2009). Functional, psychosocial and professional outcomes in long-term survivors of lower-extremity osteosarcomas: amputation versus limb salvage. Pediatr. Adolesc. Osteosarcoma.

[8] Puhaindran M.E., Chou J., Forsberg J.A., Athanasian E.A. (2012). Major upper-limb amputations for malignant tumors. J. Hand Surg..

[9] Taylor M., Pichora D. (2015). Forequarter amputation following pre-operative embolization to treat two upper extremity malignancies in a previously irradiated tissue bed: a case report. Orthop. Muscular Syst..

[10] Dimas V., Kargel J., Bauer J., Chang P. (2007). Forequarter amputation for malignant tumours of the upper extremity: Case report, techniques and indications. Can. J. Plast. Surg..

[11] Qadir R., Sidhu S., Romine L., Meyer M.S., Duncan S.F.M. (2014). Interscapulothoracic (forequarter) amputation for malignant tumors involving the upper extremity: surgical technique and case series. J. Shoulder Elb. Surg..

[12] Agha R.A., Franchi T., Sohrabi C., Mathew G., for the SCARE Group (2020). The SCARE 2020 guideline: updating consensus surgical CAse REport (SCARE) guidelines. Int. J. Surg..

[13] Suurmeijer A., Ladanyi M., Ladanyl T. (2020). Synovial sarcoma in WHO classification of tumours. Soft Tissue and Bone Tumours.

[14] Al-Busaidi S.S., Al-Hashmi S.N., Jayachandran R.K. (2017). Management of longstanding synovial sarcoma of the shoulder region – a case report. JPRAS Open.

[15] Malawer M., Sugarbaker P. (2001). Forequarter amputation. Musculoskeletal Cancer Surgery.

[16] Elsner U., Henrichs M., Gosheger G., Dieckmann R., Nottrott M., Hardes J. (2016). Forequarter amputation: a safe rescue procedure in a curative and palliative setting in high-grade malignoma of the shoulder girdle. World J. Surg. Oncol..

[17] Daigeler A., Lehnhardt M., Khadra A., Hauser J., Steinstraesser L., Langer S. (2009). Proximal major limb amputations - a retrospective analysis of 45 oncological cases. World J. Surg. Oncol..

[18] Wittig J.C., Bickels J., Kollender Y., Kellar-Graney K.L., Meller I., Malawer M.M. (2001). Palliative forequarter amputation for metastatic carcinoma to the shoulder girdle region: indications, pre-operative evaluation, surgical technique, and results. J. Surg. Oncol..

[19] Almgard L.E., Fernstrom I., Haverling M., Ljungqvist A. (1973). Treatment of renal adenocarcinoma by embolic occlusion of the renal circulation. Br. J. Urol..

[20] Nierlich P., Funovics P., Dominkus M., Aszmann O., Frey M., Klepetko W. (2011). Forequarter amputation combined with chest wall resection: a single-center experience. Ann. Thorac. Surg..

[21] Cartoni C., Niscola P., Breccia M., Brunetti G., D’Elia G.M., Giovannini M. (2009). Hemorrhagic complications in patients with advanced hematological malignancies followed at home: an Italian experience. Leuk. Lymphoma.

[22] Johnstone C., Rich S.E. (2018). Bleeding in cancer patients and its treatment: a review. Ann. Palliat. Med..

[23] Jang H.S.I., Spillane A., Boyle F., Fogarty G. (2012). Radiotherapy can cause haemostasis in bleeding skin malignancies. Case Rep. Med..

[24] Iwamoto S., Takao S., Nose H., Otomi Y., Takahashi M., Nishisho T. (2012). Usefulness of transcatheter arterial embolization prior to excision of hypervascular musculoskeletal tumors. J. Med. Invest..

[25] Exhibit S., Kartsouni V., Milatou M., Gkeli M.G. (2015). Pre-Operative Embolization of Malignant Soft Tissue Tumors of the Extremities.

[26] Jiang C., Wang J., Wang Y., Zhao J., Zhu Y., Ma X. (2016). Treatment outcome following transarterial chemoembolization in advanced bone and soft tissue sarcomas. Cardiovasc. Intervent. Radiol..

[27] van den Hout W.B., van der Linden Y.M., Steenland E., Wiggenraad R.G.J., Kievit J., de Haes H. (2003). Single- versus multiple-fraction radiotherapy in patients with painful bone metastases: cost-utility analysis based on a randomized trial. JNCI J. Natl. Cancer Inst..

[28] Yalçınkaya U., Uğraş N., Özgün G., Ocakoğlu G., Deligönül A., Çetintaş S.K. (2017). Enhancer of zeste homologue 2 (EZH2) expression in synovial sarcomas as a promising indicator of prognosis. Bosn. J. Basic Med. Sci..

[29] Cihoric N., Crowe S., Eychmüller S., Aebersold D.M., Ghadjar P. (2012). Clinically significant bleeding in incurable cancer patients: effectiveness of hemostatic radiotherapy. Radiat. Oncol..

[30] Lee J.A., Lim D.H., Park W., Ahn Y.C., Huh S.J. (2009). Radiation therapy for gastric cancer bleeding. Tumori.

[31] Sapienza L.G., Ning M.S., Jhingran A., Lin L.L., Leão C.R., da Silva B.B. (2019). Short-course palliative radiation therapy leads to excellent bleeding control: a single centre retrospective study. Clin. Transl. Radiat. Oncol..

